# Machine learning algorithms to predict heart failure with preserved ejection fraction among patients with premature myocardial infarction

**DOI:** 10.3389/fcvm.2025.1571185

**Published:** 2025-05-07

**Authors:** Jing-xian Wang, Chang-ping Li, Zhuang Cui, Yan Liang, Yu-hang Wang, Yu Zhou, Yin Liu, Jing Gao

**Affiliations:** ^1^ Clinical School of Thoracic, Tianjin Medical University, Tianjin, China; ^2^ School of Public Health, Tianjin Medical University, Tianjin, China; ^3^ Department of Cardiology, Tianjin Chest Hospital, Tianjin, China; ^4^ Chest Hospital, Tianjin University, Tianjin, China; ^5^ Cardiovascular Institute, Tianjin Chest Hospital, Tianjin, China; ^6^ Tianjin Key Laboratory of Cardiovascular Emergency and Critical Care, Tianjin, China

**Keywords:** premature myocardial infarction, heart failure with preserved ejection fraction, machine learning, XGBoost, prediction

## Abstract

**Background:**

Heart Failure with Preserved Ejection Fraction (HFpEF) in patients with Premature Myocardial Infarction (PMI) is a crucial factor affecting long-term prognosis. This study aims to develop a model based on a machine learning algorithm that can predict the risk of in-hospital HFpEF in patients with PMI early and quickly.

**Methods:**

This prospective study consecutively included PMI patients from January 2017 to December 2022. Lasso-Logistic, XGBoost, Random Forest, K-Nearest Neighbor, and Support Vector Machine models were constructed. The prediction performance of the models was compared through AUC, Accuracy, Precision, F1 score, and Brier score. Shapley Additive exPlanations is used to explain the model. A prediction system was developed to identify high-risk patients.

**Results:**

The study finally included 840 PMI patients. 268 (31.90%) developed in-hospital HFpEF. The XGBoost model has the best prediction performance (AUC 0.854; Accuracy 0.798; Precision 0.686; F1 score 0.586; Brier score 0.143). The final model included ten variables, which were Brain natriuretic peptide (BNP) > 100pg/ml, SYNTAX Score > 14.5, Age, Monocyte to Lymphocyte Ratio (MLR) > 0.3, Hematocrit (HCT) < 45%, Heart rate (HR) > 75 bpm, Body Mass Index (BMI) ≥ 24 kg/m^2^, C-reactive Protein to Lymphocyte Ratio (CLR) > 2.83, Hypertension and Fibrinogen (Fg) > 4 g/L.

**Conclusions:**

The explainable prediction model established based on the XGBoost algorithm can accurately predict the risk of in-hospital HFpEF in PMI patients and is available at https://hfpefpmi.shinyapps.io/apppredict/. This system is expected to assist clinicians in decision-making by providing timely, prioritized, and precise interventions for PMI patients, ultimately reducing the incidence of HFpEF and improving long-term prognosis.

## Introduction

The risk of heart failure (HF) remains after premature myocardial infarction (PMI) and is a determinant of poor prognosis (1). Heart Failure with Preserved Ejection Fraction (HFpEF) is defined as HF with a left ventricular ejection fraction (LVEF) ≥50%. As a result of improved treatments and increased public awareness of HFpEF, the proportion of HFpEF in HF has increased to approximately 50%, affecting up to 32 million people worldwide (2). Previous studies have shown that 30%–60% of patients with HFpEF have a history of acute myocardial infarction (AMI) (3). The in-hospital occurrence of HFpEF after AMI significantly affects the long-term prognosis, especially in the younger population, where patients suffer from decreased or even loss of their labor capacity, which in turn triggers a significant increase in the cost of medical care and creates a massive burden on the family and society (4). Therefore, a rapid, accurate, and reliable comprehensive algorithm to assess the risk in this specific group is needed for the early identification of high-risk patients with poor prognosis and for the timely improvement of long-term prognosis and quality of survival through personalized treatment.

While logistic regression (LR) remains computationally efficient and highly interpretable, it is constrained by its linear assumption, making it difficult to capture complex interactions. Additionally, LR struggles with high-dimensional data, relies heavily on manual feature selection, and is sensitive to outliers and missing values. In contrast, machine learning (ML) has gained increasing attention in cardiovascular disease prediction due to its ability to process large-scale clinical data and uncover intricate patterns (5, 6). Various ML algorithms have been widely applied to classification and predictive tasks. Among them, Extreme Gradient Boosting (XGBoost) has gained prominence for its ability to integrate multiple weak learners through a gradient boosting tree framework, achieving superior predictive performance while effectively mitigating overfitting risk (7). This ensemble approach performs exceptionally well in both binary and multiclass classification tasks and often outperforms traditional logistic regression models in clinical prediction settings (8). Random Forest (RF) is another ensemble learning method that constructs multiple decision trees and combines their outputs through averaging or majority voting, thereby enhancing model generalizability and reducing variance (9). K-Nearest Neighbors (KNN) is a simple yet effective classification algorithm that assigns class labels based on the proximity of data points (10, 11). Support Vector Machine (SVM) constructs an optimal hyperplane to maximize the margin between different classes, making it well-suited for binary classification tasks in structured datasets (12). Previous studies have demonstrated that XGBoost excels in predicting 30-day readmission in HF patients (13), AMI outcomes (14), ICU mortality (15), and acute kidney injury (16), whereas Random Forest has shown superior performance in postoperative delirium prediction (17).

Despite numerous advances, few applications of machine learning have been addressed in predicting HFpEF events during hospitalisation in patients with PMI. Given that PMI patients may exhibit unique risk factors, this study leverages the strengths of multiple ML algorithms to develop a predictive model integrating multidimensional clinical features of PMI patients. Specifically, we aim to identify key predictive factors, quantify their contributions, and provide a foundation for risk stratification and early intervention in this high-risk cohort.

## Material and methods

### Study population

**Figure 1:**
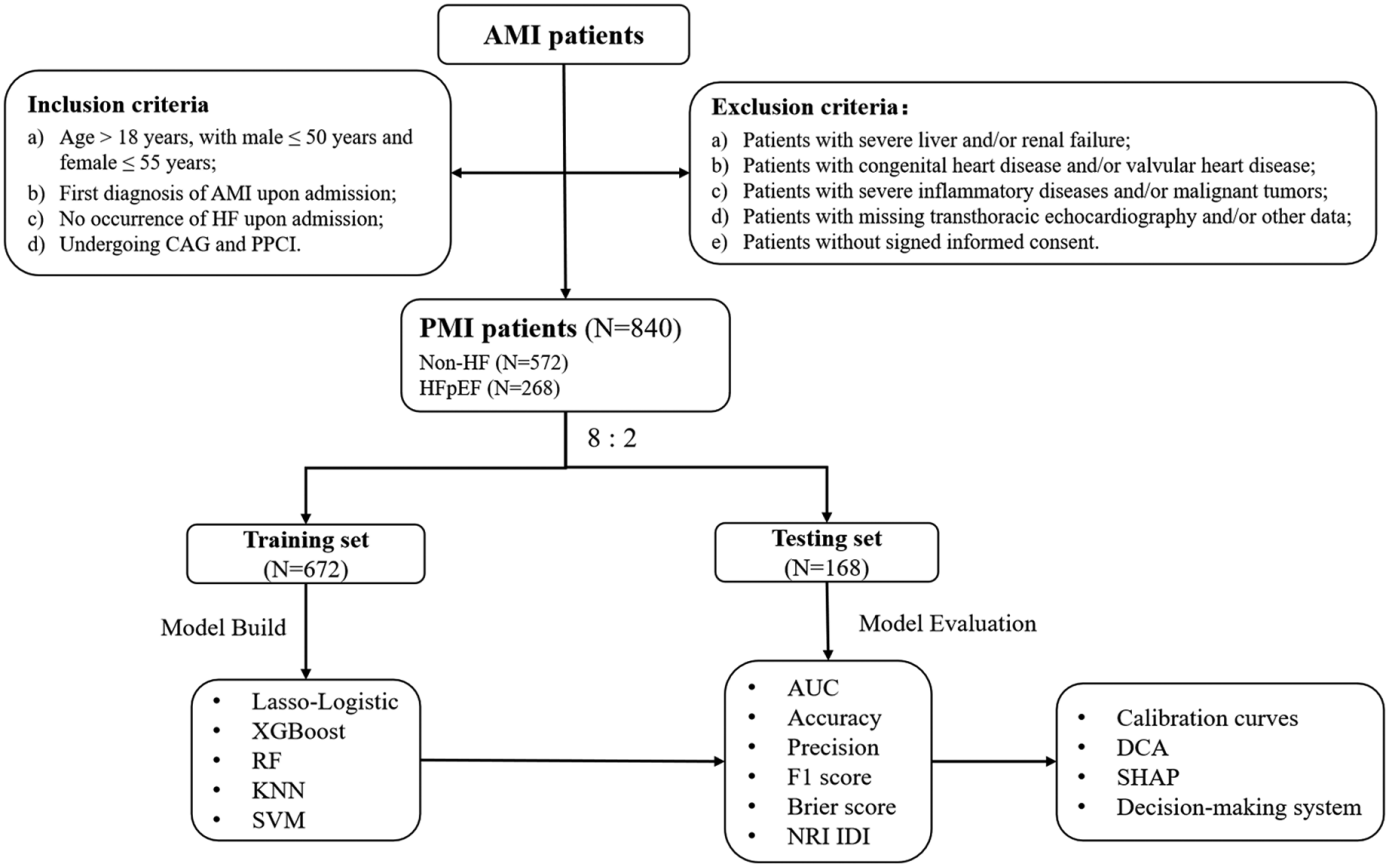
Flow chart of the study. AMI, acute myocardial infarction; CA. G, coronary angiography; DCA, decision curve analysis; HF, heart failure; HFpEF, heart failure with preserved ejection fraction; PPCI, primary percutaneous coronary intervention; PMI, premature myocardial infarction; SHAP: shapley additive exPlanations; NRI, net reclassification improvement; IDI, integrated discriminant improvement.

The flow of the study is shown in Figure 1. This is a single-center, prospective, observational cohort study. Consecutive patients admitted to Tianjin Chest Hospital for AMI between January 2017 and December 2022, meeting the PMI age threshold, were included in the PMI cohort. The inclusion criteria were as follows: (a) Age > 18 years, with male ≤ 50 years and female ≤ 55 years; (b) First diagnosis of AMI upon admission, meeting the universal definition including clinical symptoms, typical changes in the electrocardiogram, and elevated cardiac biomarkers (18, 19). (c) No occurrence of HF upon admission; (d) Undergoing coronary angiography (CAG) and primary percutaneous coronary intervention (PPCI). CAG and PPCI were performed by two or more cardiologists qualified in coronary diagnosis and treatment at our center.

The exclusion criteria were as follows: (a) Patients with severe liver and/or renal failure; (b) Patients with congenital heart disease and/or valvular heart disease; (c) Patients with severe inflammatory diseases and/or malignant tumors; (d) Patients with missing transthoracic echocardiography and/or other data; (e) Patients without signed informed consent. This study was conducted in accordance to the Declaration of Helsinki and was approved by the Ethics Committee of Tianjin Chest Hospital (No. 2017KY-007-01).

### Data collection

We developed a structured electronic medical record database specifically for PMI patients at our hospital. This database was designed to standardize data collection and facilitate analysis. The collected data includes age, sex, body mass index (BMI), smoking and alcohol habits, family history of coronary artery disease (CAD), previous history, type of AMI; admission vital signs; laboratory examinations, CAG, and transthoracic echocardiography (TTE) parameters. TTE was conducted by certified sonographers following a standardized image acquisition protocol and utilizing consistent imaging equipment to capture the final result before discharge. Inflammatory biomarkers were determined using the following formulas, CLR (C-reactive Protein (mg/L) to Lymphocyte (*10^9^/L) ratio) and MLR (Monocyte (*10^9^/L) to Lymphocyte (*10^9^/L) ratio). The SYNTAX Score (20) was utilized to evaluate the severity of CAD and to aid in risk stratification and the planning of revascularization strategies among patients with CAD. It was calculated using software available online (https://syntaxscore.org/). In addition, we recorded the patient's medication status during hospitalization, including antiplatelet drugs, statins, diuretics, angiotensin-converting enzyme inhibitors (ACEI), angiotensin-receptor blockers (ARB), angiotensin receptor-neprilysin inhibitor (ARNI), and beta-blockers.

### Study endpoint

All patients had a TTE assessment before discharged. HF was defined according to the criteria established by the guidelines for HF (21, 22). The diagnostic criteria for HFpEF are as follows:

(1) Presence of at least one typical heart failure symptom (e.g., exertional dyspnea, orthopnea) AND/OR one clinical sign of fluid retention (e.g., jugular venous distension, pulmonary rales, Leg edema).
(2) Objective evidence of cardiac congestion demonstrated by either:
  (a) Hemodynamic confirmation *via* right heart catheterization [resting pulmonary capillary wedge pressure (PCWP)>15 mmHg],
  (b) tructural/functional abnormalities through imaging: Thoracic radiography demonstrating pulmonary vascular redistribution or interstitial edema; Echocardiographic indices elevated *E*/*e*′ ratio (>13).
(3) Echocardiography indicates that the LVEF is ≥50%.

### Model construction and validation

The minimum sample size required to construct the predictive model based on the pmsampsize criterion proposed by Riley et al. (23) requires the inclusion of at least 298 subjects. The missing rate of each variable was less than 5%; we used the median to fill in the missing values for continuous variables and conducted multiple imputations for categorical variables. The parameters for multiple imputation were set as m = 5, method = NULL, maxit = 50, and seed = 123. The number of missing values for each variable and the analysis results of the dataset before and after imputation are presented in Supplementary Table S1. To construct the model, the dataset is first randomly divided into: 80% as a training set for model training and cross-validation, and 20% as a testing set for final model evaluation. Within the training set, a five-fold cross-validation is further employed. The training data is randomly partitioned into five equal folds. For each iteration, four folds are selected for training, and one fold is used for validation. This process is repeated five times to optimize hyperparameters and reduce the risk of overfitting. The final model is evaluated using the 20% testing set to ensure a true assessment of its generalization performance. Considering the wide range of intervals for the different variables, as well as achieving the goal of rapid clinical application of the prediction system, we converted continuous variables other than age into categorical variables for analysis based on reference ranges, guidelines, or medians, and normalized age by a min-max scaling. Specifically, BMI was classified according to the Chinese population's overweight standard (24 kg/m^2^) (24). Heart rate (HR), systolic blood pressure (SBP), diastolic blood pressure (DBP), white blood cell count (WBC), neutrophils percentage (NEU%), hematocrit (HCT), hemoglobin (Hb), platelet count (PLT), total bilirubin (TBil), creatinine (Cr), uric acid (UA), homocysteine (HCY), glucose (Glu), glycated hemoglobin (HbA1c), total cholesterol (TC), triglycerides (TG), low-density lipoprotein cholesterol (LDL-C), high-density lipoprotein cholesterol (HDL-C), free fatty acids (FFA), C-reactive protein (CRP), D-dimer, fibrinogen (Fg), and brain natriuretic peptide (BNP) were classified according to their normal value reference ranges or guideline recommendations for classification. We classified total bile acids (TBA), CLR, MLR, creatine kinase isoenzyme (CK-MB), cardiac troponin T (cTnT), and SYNTAX scores according to the median of the dataset due to the lack of clear classification criteria. The number of HFpEF patients in this study is lower than that of non-HF patients. This imbalance in the data samples could significantly impact the performance of the prediction model. Therefore, we use the Synthetic Minority Over-sampling Technique (SMOTE) to balance the data set in the training set. The k-Nearest Neighbor (k-NN) threshold used in SMOTE is 5, and the over-sampling ratio is 1. This technique involves increasing the number of samples in the minority class by synthesizing new samples. Achieving a balanced number of samples in the data set is crucial for improving the model's training effectiveness (25).

Five ML models, Least absolute shrinkage and selection operator (Lasso)-Logistic, RF, XGBoost, KNN, and SVM, were constructed using the data from the training set with whether or not an in-hospital HFpEF occurred as the outcome event. Gradient boosting technology is based on decision trees. Variance inflation factor (VIF) was used to assess multicollinearity among predictor variables, and variables with VIF greater than 10 were excluded. Lasso is a regularization technique that performs variable selection and coefficient estimation by imposing constraints on the sum of the absolute values of the model parameters. This process causes some coefficients to be reduced to zero, effectively excluding them from the final model. Using the variables screened by Lasso, a two-category logistic risk prediction model is constructed. The modeling process of the other four models (RF, XGBoost, KNN, and SVM) is as follows. First, all variables are trained. To optimize the model's performance, the automatic parameter adjustment strategy is adopted, and a 5-fold cross-validation is used as the re-sampling strategy to evaluate the model's generalization ability. To further optimize model performance, we rank the variables based on their importance and select the top 10 features for retraining, following the same procedure as described above.

### Statistical analysis

Shapiro–Wilk test was used to evaluate the normality of continuous variables. Mean ± standard deviation (SD) or median (interquartile) was used to describe variables according to the evaluation results. *T*-test or Mann–Whitney test was used for inter-group comparison. The categorical variables were expressed as numbers and percentages, and the chi-square test or Fisher's exact test was used for intergroup comparisons.

To evaluate the models, the area under the curve (AUC), Precision, Accuracy, F1 score, and Brier score were calculated in the testing set. Net Reclassification Improvement (NRI) and Integrated Discriminant Improvement (IDI) are used to assess the enhancement of the predictive model. According to the comparison results, the model with the best comprehensive performance was selected to construct the in-hospital HFpEF prediction model of PMI patients. The final model is explained using Shapley Additive exPlanations values (SHAP). Calibration curves determine how close the model's predicted probabilities are to the actual observed probabilities. Decision Curve Analysis (DCA) quantifies the net benefit of using the model at different thresholds and assesses the utility of the model in decision-making. Finally, a visual online prediction system was constructed to calculate the prediction probability for clinical application.

All statistical analyses were performed in SPSS Statistics 26.0 (IBM, Chicago, USA) and R software (version 4.3.1). Specifically, SPSS was used for descriptive statistics (normality tests, means, standard deviations and frequency distributions), and basic inferential analyses (*t*-tests, one-way ANOVA, and chi-square tests). R was employed for model construction, validation, performance evaluation and visualization analysis. A two-sided *P* < 0.05 was considered statistically significant.

## Results

### Baseline characteristics

The baseline characteristics of the study population, stratified into the HFpEF group (*n* = 268) and the Non-HF group (*n* = 572), are presented in Table 1. The total cohort comprised 840 participants, with a median age of 42[38–44] years and a pronounced male predominance (91.0% males vs. 9.0% females). Notably, significant differences were observed between the two groups. The HFpEF group had a higher proportion of females compared to the Non-HF group (15.7% vs. 5.9%, *P* < 0.001) and a slightly older median age (42 [39–45] vs. 41 [37–44] years, *P* = 0.001). Additionally, participants with HFpEF exhibited a higher prevalence of obesity (BMI ≥ 24 kg/m^2^: 82.5% vs. 74.5%, *P* = 0.013) and hypertension (56.3% vs. 45.8%, *P* = 0.006). Laboratory and clinical findings further distinguished the groups. The HFpEF group demonstrated elevated markers of inflammation (CRP >5.0 mg/L: 63.4% vs. 43.5%, *P* < 0.001; MLR >0.3: 65.7% vs. 42.7%, *P* < 0.001) and cardiac dysfunction (BNP >100 pg/ml: 91.0% vs. 62.4%, *P* < 0.001). Coronary angiography revealed more severe coronary artery disease in HFpEF patients, as evidenced by a higher SYNTAX Score >14.5 (64.2% vs. 43.0%, *P* < 0.001).

**Table 1:** Baseline characteristics of the hFpEF group and Non-HF group.

Variables	Total (*n* = 840)	Non-HF (*n* = 572)	HFpEF (*n* = 268)	*P*
Sex, *n* (%)				<0.001
Female	76 (9.0)	34 (5.9)	42 (15.7)	
Male	764 (91.0)	538 (94.1)	226 (84.3)	
Age (years) Median (Q1, Q3)	42 (38, 44)	41 (37, 44)	42 (39, 45)	0.001
BMI ≥ 24 kg/m^2^, *n* (%)	647 (77.0)	426 (74.5)	221 (82.5)	0.013
Smoke, *n* (%)	597 (71.1)	417 (72.9)	180 (67.2)	0.104
Alcohol, *n* (%)	322 (38.3)	219 (38.3)	103 (38.4)	1.000
Family history of CAD, *n* (%)	104 (12.4)	75 (13.1)	29 (10.8)	0.408
Previous history				
Hypertension, *n* (%)	413 (49.2)	262 (45.8)	151 (56.3)	0.006
Diabetes, *n* (%)	196 (23.3)	132 (23.1)	64 (23.9)	0.866
Hyperlipidemia, *n* (%)	221 (26.3)	155 (27.1)	66 (24.6)	0.500
Type of AMI, *n* (%)				0.003
NSTEMI	220 (26.2)	168 (29.4)	52 (19.4)	
STEMI	620 (73.8)	404 (70.6)	216 (80.6)	
Admission vital signs				
HR > 75 bpm, *n* (%)	98 (11.7)	51 (8.9)	47 (17.5)	<0.001
SBP > 140 mmhg, *n* (%)	832 (99.1)	567 (99.1)	265 (98.9)	0.715
DBP > 90 mmhg, *n* (%)	182 (21.7)	123 (21.5)	59 (22.0)	0.938
Laboratory examination				
WBC > 10*10^9^/L, *n* (%)	389 (46.3)	254 (44.4)	135 (50.4)	0.123
NEU% > 75%, *n* (%)	326 (38.8)	196 (34.3)	130 (48.5)	<0.001
HCT < 45%, *n* (%)	555 (66.1)	352 (61.5)	203 (75.8)	<0.001
Hb < 120 g/L, *n* (%)	33 (3.9)	14 (2.5)	19 (7.1)	0.002
PLT > 300*10^12^/L, *n* (%)	136 (16.2)	89 (15.6)	47 (17.5)	0.532
TBA > 1.56 umol/L, *n* (%)	395 (47.0)	271 (47.4)	124 (46.3)	0.821
TBil > 17.1 umol/L, *n* (%)	229 (27.3)	145 (25.4)	84 (31.3)	0.083
Cr > 110 umol/L, *n* (%)	22 (2.6)	11 (1.9)	11 (4.1)	0.107
UA > 360 umol/L, *n* (%)	392 (46.7)	279 (48.8)	113 (42.2)	0.086
HCY > 15 umol/L, *n* (%)	280 (33.3)	191 (33.4)	89 (33.2)	1.000
Glu > 7.0 mmol/L, *n* (%)	212 (25.2)	140 (24.5)	72 (26.9)	0.510
HbA1c > 6.5%, *n* (%)	176 (21.0)	118 (20.6)	58 (21.6)	0.806
TC > 5.20 mmol/L, *n* (%)	283 (33.7)	193 (33.7)	90 (33.6)	1.000
TG > 1.70 mmol/L, *n* (%)	525 (62.5)	362 (63.3)	163 (60.8)	0.541
LDL-C > 3.40 mmol/L, *n* (%)	339 (40.4)	225 (39.3)	114 (42.5)	0.420
HDL-C < 1.0 mmol/L, *n* (%)	593 (70.6)	414 (72.4)	179 (66.8)	0.115
FFA > 0.9 mmol/L, *n* (%)	65 (7.7)	39 (6.8)	26 (9.7)	0.187
CRP > 5.0 mg/L, *n* (%)	419 (49.9)	249 (43.5)	170 (63.4)	<0.001
CLR > 2.83, *n* (%)	420 (50.0)	246 (43.0)	174 (64.9)	<0.001
MLR > 0.3 *n* (%)	420 (50.0)	244 (42.7)	176 (65.7)	<0.001
D-dimer > 0.5 mg/L, *n* (%)	110 (13.1)	240 (42.0)	168 (62.7)	<0.001
Fg > 4.0 g/L, *n* (%)	161 (19.2)	92 (16.1)	69 (25.8)	0.001
CK-MB > 77 U/L, *n* (%)	413 (49.2)	259 (45.3)	154 (57.5)	0.001
cTnT > 1.44 ng/L, *n* (%)	420 (50.0)	255 (44.6)	165 (61.6)	<0.001
BNP > 100 pg/ml, *n* (%)	601 (71.6)	357 (62.4)	244 (91.0)	<0.001
Coronary angiography				
SYNTAX Score > 14.5, *n* (%)	418 (49.8)	246 (43.0)	172 (64.2)	<0.001
Three-vessel artery disease, *n* (%)	255 (30.4)	169 (29.6)	86 (32.1)	0.505
Left main artery disease, *n* (%)	19 (2.3)	9 (1.8)	10 (3.7)	0.087
Complete coronary occlusion, *n* (%)	452 (53.8)	301 (52.6)	151 (56.3)	0.350
LVEF (%)	55 (51, 58)	55 (50, 59)	55 (52, 58)	0.943
Symptoms and signs				
Jugular venous distension	117 (13.93)	0 (0.00)	117 (43.95)	<0.001
Pulmonary rales	102 (12.14)	0 (0.00)	102 (38.06)	<0.001
Leg edema	58 (6.90)	0 (0.00)	88 (21.64)	<0.001
Killip class				<0.001
I	792 (94.29)	572 (100.00)	220 (82.09)	
II	43 (5.12)	0 (0.00)	43 (16.04)	
III	5 (0.60)	0 (0.00)	5 (1.87)	
IV	0 (0.00)	0 (0.00)	0 (0.00)	
Medication during hospitalization				
Aspirin	840 (100.00)	572 (100.00)	268 (100.00)	–
P2Y12 inhibitors	840 (100.00)	572 (100.00)	268 (100.00)	–
Statins	830 (98.81)	563 (98.43)	267 (99.63)	0.135
Beta-blockers	645 (76.69)	211 (78.73)	434 (75.87)	0.361
ACEI/ARB	561 (66.79)	379 (66.26)	182 (67.91)	0.636
ARNI	93 (11.07)	30 (11.19)	63 (11.01)	0.938
Loop diuretics	30 (3.57)	0 (0.00)	30 (11.19)	<0.001
MRA	41(4.88)	0(0.00)	41(15.30)	<0.001

ACEI, angiotensin-converting enzyme inhibitor; ARB, angiotensin-receptor blocker; ARNI, angiotensin receptor-neprilysin inhibitor; BMI, body mass index; BNP, brain natriuretic peptide; CAD, coronary artery disease; CK-MB, creatine kinase isoenzyme; CLR, C-reactive protein to lymphocyte ratio; cTnT, cardiac troponin T; Cr, creatinine; CRP, C-reactive protein; DBP, diastolic blood pressure; FFA, free fatty acids; Fg, fibrinogen; Glu, glucose; Hb, hemoglobin; HbA1c, glycated hemoglobin; HCT, hematocrit; HCY, Homocysteine; HDL-C, high-density lipoprotein cholesterol; HR, heart rate; LDL-C, low-density lipoprotein cholesterol; LVEF, left ventricular ejection fraction; MLR, monocyte to lymphocyte ratio; MRA, mineralocorticoid receptor antagonist; NEU%, neutrophils percentage; NSTEMI, non-ST-segment elevation myocardial infarction; PLT, platelet; SBP, systolic blood pressure; STEMI, ST-segment elevation myocardial infarction; TBA, total bile acids; TBil, total bilirubin; TC, total cholesterol; TG, triglyceride; UA, uric acid; WBC, white blood cell.

In summary, the HFpEF cohort was characterized by older age, a higher burden of comorbidities, and more pronounced cardiovascular dysfunction compared to the Non-HF group.

Supplementary Table S2 shows the baseline characteristics of the training set and testing set. Supplementary Table S3 shows the descriptive statistics of continuous variables.

### Model construction

According to the ratio of 8:2, 840 patients were randomly divided into a training set and a testing set, with 672 in the training set and 168 in the testing set, and five different models were constructed in the training set. The VIF values are all below 5, indicating that multicollinearity is not a significant issue in our model.

**Figure 2:**
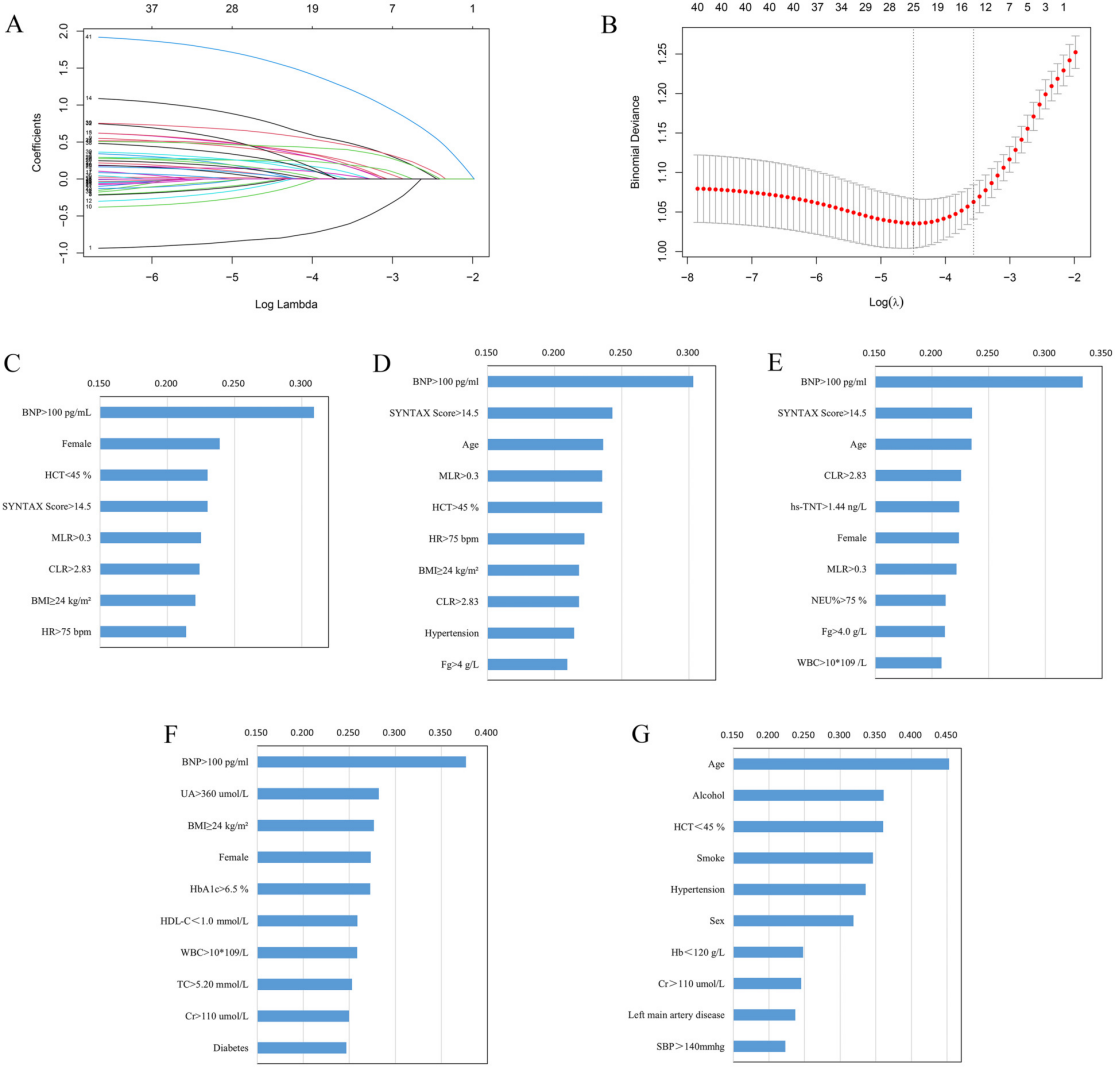
**(A)** Lasso regression coefficients vs. *λ* values; **(B)** cross-validated *λ* and classification error plot relationships. Ranking of important variables based on Lasso-Logistic **(C)**, XGBoost **(D)**, RF **(E)**, KNN **(F)**, and SVM **(G)** models. BMI, body mass index; BNP, brain natriuretic peptide; CLR, C-reactive protein to lymphocyte ratio; cTnT, cardiac troponin T; Cr, creatinine; Fg, fibrinogen; Hb, hemoglobin; HbA1c, glycated hemoglobin; HCT, hematocrit; HDL-C, high-density lipoprotein cholesterol; HR, heart rate; MLR, monocyte to lymphocyte ratio; NEU%, neutrophil percentage; SBP, systolic blood pressure; TC, total cholesterol; UA, uric acid; WBC, white blood cell.

Lasso regression screening (Figures 2A,B) was performed on the full set of variables, identifying 12 key predictors: female sex, hypertension, STEMI, BMI ≥24 kg/m^2^, SYNTAX Score >14.5, admission HR >75 bpm, NEU% >75%, HCT <45%, cTnT >1.44 ng/L, MLR >0.3, CLR >2.83, and BNP >100 pg/ml. Multivariate logistic regression modeling was carried out using the 12 variables from the screening. The final predictive model obtained consisted of 8 variables, which were female, BMI ≥24 kg/m^2^, SYNTAX Score >14.5, admission HR >75 bpm, HCT <45%, MLR >0.3, CLR >2.83, and BNP >100 pg/ml.

Following model training using RF, XGBoost, KNN, and SVM, the top 10 most important variables were identified and used for subsequent modeling. The ordering of important variables based on the Lasso-Logistic, XGBoost, RF, KNN, and SVM models are shown in Figures 2C–G, respectively.

### Model performance

**Figure 3:**
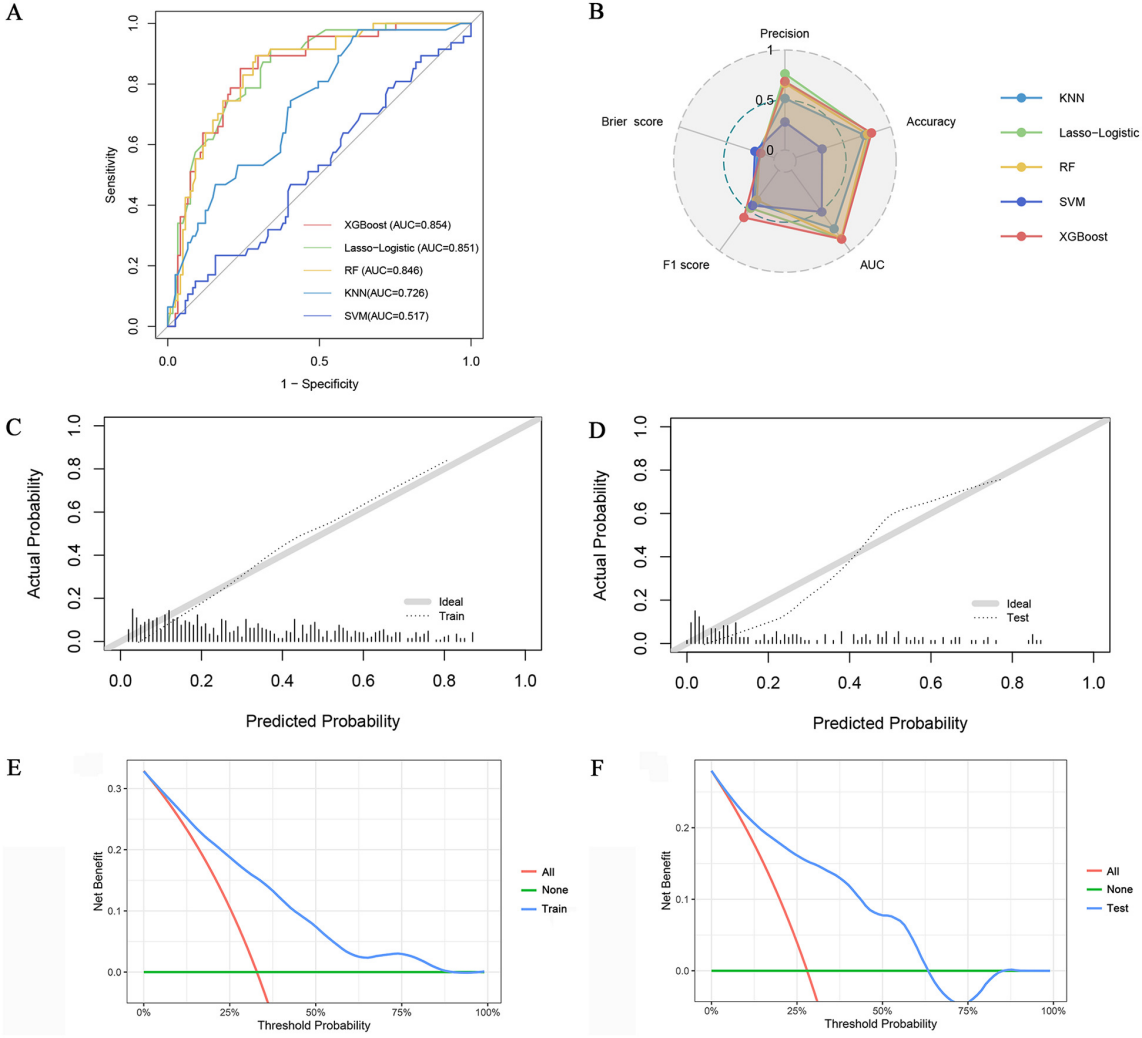
**(A)** ROC curve of five algorithms; **(B)** Radar plot of precision, accuracy, AUC, F1 score, and Brier score of the five models; Calibration curves of the XGBoost model in the training set **(C)** and testing set **(D)** DCA of the XGBoost model in the training set **(E)** and testing set **(F)**.

The classification performance of different models is compared in the testing set. (Table 2, Figures 3A,B). Regarding discrimination, the AUC values of the five models range from 0.517–0.854, with the highest being XGBoost and the lowest being SVM. The F1 score for combined precision and recall was the highest among the XGBoost models (0.586). A radar plot was made based on precision, accuracy, AUC, F1 score, and Brier score, which reflects the performance of each model (Figure 3B). Although the XGBoost model predicts a lower accuracy than Lasso-Logistic (0.686 vs. 0.762), its AUC, accuracy, and F1 score are the highest, and its Brier score is comparable to Lasso-Logistic (0.143 vs. 0.143). Supplementary Figure S1 illustrates the class distribution of the training set before and after applying the SMOTE. The figure demonstrates that SMOTE effectively addresses class imbalance by generating synthetic samples for the minority class, ensuring a more balanced distribution for model training. We noticed improvements in the performance of most models, with the exception of SVM. In particular, the F1 score of the combined precision and recall, and the AUC of the XGBoost and KNN models also improved. Even after balancing the classes with SMOTE, XGBoost remained the best-performing model (Supplementary Table S4). Supplementary Table S5 shows the NRI and IDI calculated for the XGBoost and four other models. The NRI calculated by the XGBoost model and Lasso-logistic was 0.149 (95% CI: 0.008–0.290, *P* = 0.039), and the IDI was 0.049 (95% CI: 0.008–0.091, *P* = 0.019). The NRI calculated by the XGBoost model and RF model was 0.222 (95% CI: 0.067–0.377, *P* = 0.005), and the IDI was 0.051 (95% CI: 0.015–0.086, *p* = 0.005). The NRI calculated by the XGBoost model and KNN model was 0.212 (95% CI: 0.028–0.396, *P* = 0.025), and the IDI was 0.067 (95% CI: 0.004–0.130, *p* = 0.038). The NRI calculated with the XGBoost model and the SVM model was 0.428 (95%CI: 0.277–0.579, *P* < 0.001), and the IDI was 0.227 (95% CI: 0.175–0.279, *P* < 0.001). These results indicate that the XGBoost model demonstrates stronger predictive ability compared to the other models.

**Table 2:** Classification performance of five models on the testing set.

Model	Precision	Accuracy	AUC	F1 score	Brier score
Lasso-Logistic	0.762	0.786	0.851	0.470	0.143
XGBoost	0.686	0.798	0.854	0.586	0.143
RF	0.667	0.756	0.846	0.369	0.155
KNN	0.516	0.726	0.726	0.410	0.177
SVM	0.280	0.280	0.517	0.438	0.204

Calibration curves show that the predicted probabilities of the XGBoost model are in good agreement with the actual probabilities (Figures 3C,D). The DCA plot (Figures 3E,F) demonstrates that the XGBoost model provides positive net benefits across a range of threshold probabilities in both the training and testing sets. This suggests that the model has strong clinical utility in aiding clinicians with accurate risk assessment and decision-making for PMI patients. In conclusion, the XGBoost model was selected as the final model based on its superior performance.

### Importance ranking

**Figure 4:**
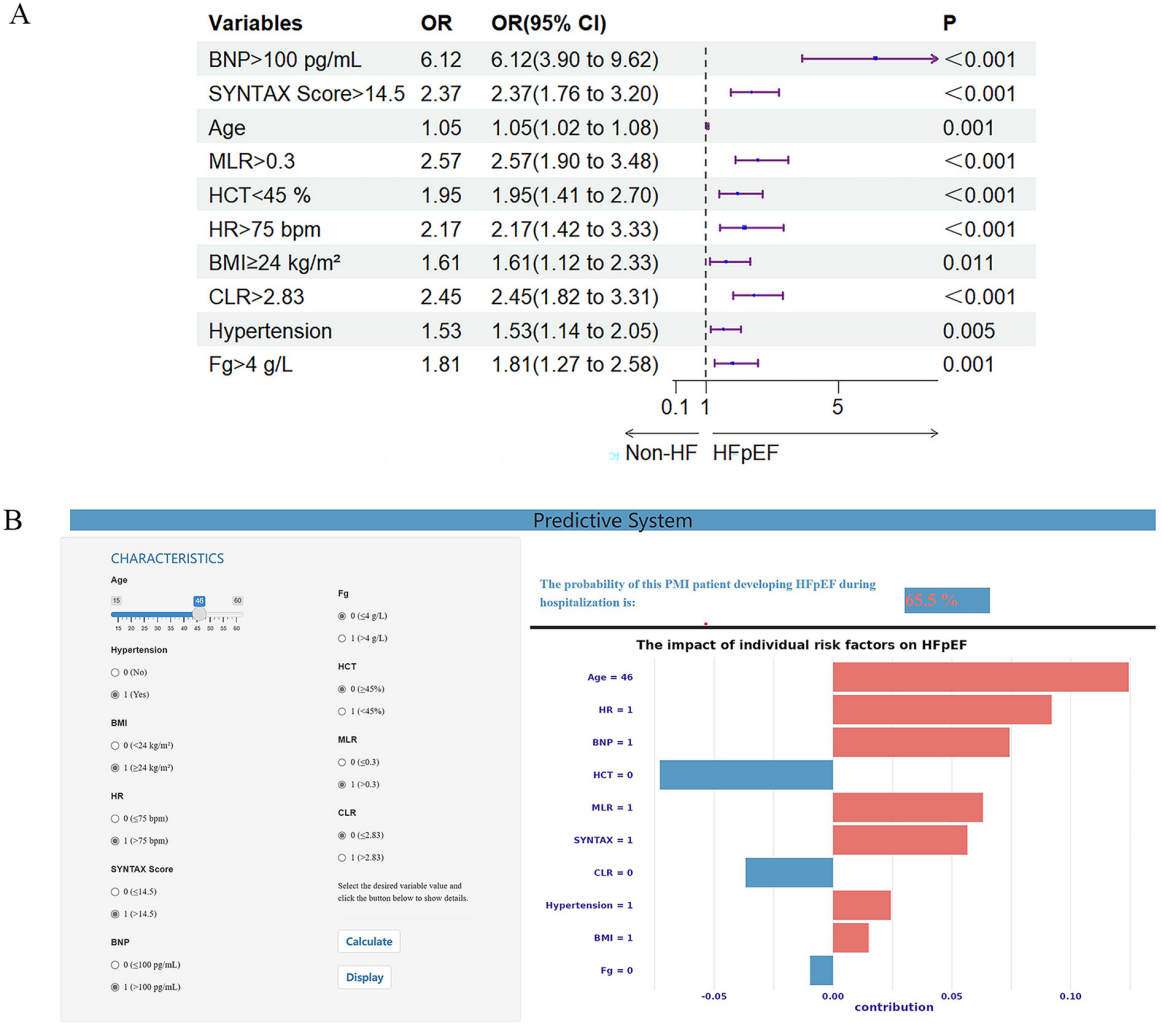
**(A)** Forest plot with 10 important variables based on the XGBoost model. **(B)** Visual online prediction system based on the XGBoost model. The input interface is on the left, and the output interface is on the right. The upper part of the right represents the prediction probability, and the lower part of the right shows an individualized SHAP diagram. The red feature makes the model recognize the sample as class 1, while the blue feature makes the model identify the sample as class 0. BMI, body mass index; BNP, brain natriuretic peptide; CLR, C-reactive protein to lymphocyte ratio; Fg, fibrinogen; HCT, hematocrit; HF, heart failure; HFpEF, heart failure with preserved ejection fraction; HR, heart rate; MLR, monocyte to lymphocyte ratio.

The XGBoost model's variables' importance ranking showed that BNP >100 pg/ml is the most important characteristic influencing the occurrence of in-hospital HFpEF in patients with PMI. In addition, SYNTAX Score >14.5, Age, MLR >0.3, HCT <45%, HR >75 bpm, BMI ≥24 kg/m^2^, CLR >2.83, Hypertension, and Fg ≥4 g/L were also important variables in predicting HFpEF (Figure 2D). Figure 4A and Supplementary Table S6 shows that the above variables were risk factors for in-hospital HFpEF in patients with PMI.

### Visual prediction system

The visual prediction system includes an input interface (left) for entering patient variables (e.g., age, hypertension status) and an output interface (right) displaying predicted probabilities (top) and individualized SHAP charts explaining model decisions (bottom). In Figure 4B, the system predicts a 65.5% probability of HFpEF for a PMI patient based on clinical inputs. The system is publicly accessible at https://hfpefpmi.shinyapps.io/apppredict/.

## Discussion

In this study, we explored five ML methods to build a prediction model for HFpEF in PMI patients based on clinically accessible data. The model developed by XGBoost has superior prediction performance and can better predict whether HFpEF occurs in the hospital in patients with PMI. Based on the ranking of variable importance, the final model includes ten predictive variables: BNP > 100 pg/ml, SYNTAX Score >14.5, Age, MLR >0.3, HCT <45%, HR >75 bpm, BMI ≥24 kg/m^2^, CLR >2.83, Hypertension, and Fg >4 g/L. In addition, establishing a visual online prediction system will facilitate a more convenient clinical application, which can calculate the prediction probability of in-hospital HFpEF in PMI patients. As a model explanation, SHAP can show how each variable affects the model prediction and the degree of influence.

To our knowledge, this is the first study to develop an in-hospital HFpEF prediction model for PMI patients using ML algorithms. Previous studies, such as Liang et al. (26) and Xu et al. (27), primarily relied on logistic regression models to identify risk factors for HF in AMI patients. While logistic regression offers advantages in model interpretability and statistical rigor, it struggles to capture complex nonlinear interactions, potentially underestimating the influence of key variables. Given the highly heterogeneous pathophysiology of HFpEF, this study employs the XGBoost algorithm, which excels in identifying nonlinear relationships and intricate variable interactions, making it particularly suitable for HFpEF prediction. Our model incorporates multidimensional predictive factors, including SYNTAX score to assess coronary complexity, inflammatory markers (CLR, MLR), metabolic risk factors (BMI, hypertension), and dynamic biomarker thresholds (e.g., BNP >100 pg/ml), enabling more refined risk stratification. Unlike previous methods that primarily relied on static OR values, we implemented SHAP-based visualization analysis, which enhances model transparency and mitigates the “black-box” issue commonly associated with ML applications in clinical practice.

In recent years, Li et al. (28) demonstrated the potential of machine learning in predicting HF after AMI. Li et al. compared seven ML algorithms and identified XGBoost as the best-performing model (AUC = 0.966). However, their study primarily focused on general HF prediction rather than specifically addressing HFpEF as a distinct subtype. In contrast, our study is dedicated to HFpEF risk prediction, filling a critical gap in early risk stratification for PMI patients. Additionally, we developed an interpretable and real-time visualization tool, which can assist clinicians in rapidly assessing HFpEF risk in clinical practice. However, unlike Li et al. (28) which conducted external validation using an independent patient cohort, our study has not yet undergone multi-center external validation. Future research should focus on evaluating the model's stability and generalizability across different hospitals and populations, ensuring its clinical applicability and scalability.

The epidemiology of HF after AMI has undergone significant changes over the past decades, with an increased proportion of HFpEF and a mortality rate comparable to that of patients with Heart Failure with Reduced Ejection Fraction (HFrEF) (29). HFpEF is a heterogeneous systemic disease in which risk factors such as aging, obesity, hypertension, and systemic metabolic disorders may impair cardiac, pulmonary, vascular, and peripheral reserve capacity, abnormalities not apparent in the resting state (30). Damage and necrosis of cardiomyocytes after AMI can lead to impaired systolic reserve, and changes in cardiomyocyte and interstitial structure, such as cardiomyocyte hypertrophy and increased collagen fiber content, can lead to impaired diastolic reserve (31, 32).

Doshi et al. (33) studied the characteristics of HFpEF occurring during hospitalization in STEMI patients undergoing PCI and showed that older age, a higher proportion of females, and more comorbidities were risk factors for HFpEF. The SYNTAX Score, which reflects the extent of coronary artery disease, is also an essential factor in HFpEF. Complex coronary artery disease indicates the presence of a more significant atherosclerotic burden. The prevalence of HFpEF is strongly associated with metabolic syndrome epidemics, and obesity may be a significant driver of HFpEF in young people (34). The prevalence of subclinical left ventricular dysfunction has been confirmed to occur in Asians at lower BMI thresholds (35). In this study, HFpEF was predicted in young AMI patients with a BMI ≥24 kg/m^2^. In addition to the traditional belief that hypertension leads to increased left ventricular afterload, causing left ventricular hypertrophy and subsequent left ventricular diastolic dysfunction, the pro-inflammatory state resulting from systemic hypertension also promotes the development of HFpEF (36). HFpEF is also manifested by elevated circulating inflammatory biomarkers, and the results of the present study suggest that inflammatory markers such as CLR, MLR, and Fg. On the other hand, the increase of Fg indicates increased clotting ability and impaired blood flow. Additionally, our study demonstrates a significant gender disparity, with a markedly higher prevalence of HFpEF among PMI patients compared to males (15.7% vs. 5.9%, *p* < 0.001). This finding is consistent with existing literature on sex-specific comorbidity burden and adverse outcomes following AMI in young women (37, 38). It highlights the need for early identification and gender-specific management strategies to improve outcomes in this vulnerable population.

Combined with the predictive factors discussed in this study, for high-risk patients, first-line treatment includes early use of sodium-glucose cotransporter type 2 (SGLT2) inhibitors for patients without contraindications and diuretics for patients with evident hyperemia to maintain average blood volume (2). Revascularization treatment should be carried out for patients with complex vascular lesions as soon as possible, and related risk factors should be controlled. Patients should insist on taking medicine, limit sodium, calorie, and fluid intake, control blood pressure, and monitor BNP and inflammatory markers.

In this study, we investigated for the first time the risk factors and characteristics of in-hospital HFpEF in young patients with AMI and developed a prediction model. With the prevalence of metabolic syndrome in young people, the incidence of HFpEF is increasing, and the prognosis is poor; more attention should be given to the young population. In addition, the XGBoost algorithm can enhance the performance of the prediction model, and there has not been a previous attempt to apply the ML algorithm to predict the risk of in-hospital HFpEF in patients with PMI. This study has several limitations. First, the single-center design may limit generalizability despite internal cross-validation. Second, although the sample size was sufficient for preliminary model development, a larger cohort is necessary to validate feature stability and improve model robustness. The limited sample size might affect model robustness. Third, future studies should incorporate additional clinical dimensions including lifestyle factors (e.g., diet, physical activity), medication histories, socioeconomic parameters, and genetic profiles to improve predictive performance. Finally, external validation across diverse populations remains essential before clinical implementation.

## Conclusions

In this study, a new model was developed to predict the risk of in-hospital HFpEF in PMI patients. In a comprehensive comparison, the XGBoost model had the best predictive ability. The XGBoost-based visual prediction system shows clinical decision-support potential for PMI management, pending rigorous external validation in diverse clinical settings. Following successful validation, it could provide early and precise intervention guidance for PMI patients to reduce HFpEF incidence and improve long-term prognosis.

## Data Availability

The original contributions presented in the study are included in the article/Supplementary Material, further inquiries can be directed to the corresponding authors.
