# Supplementary material for: Machine learning algorithms to predict heart failure with preserved ejection fraction among patients with premature myocardial infarction
Source: Front Cardiovasc Med. 2025 May 7;12:1571185. doi: 10.3389/fcvm.2025.1571185 (PMC12092383; doi:10.3389/fcvm.2025.1571185)
Supplement: Supplementary file 1 [file Table1.docx]

**Table S1.** Comparative Analysis Before and After Variable imputation

| **Variables** | **Number of missing values** | **Before imputation** | **After imputation** | ***P*** |
| --- | --- | --- | --- | --- |
| BMI, M (Q₁, Q₃) | 20 | 26.00 (24.22, 27.78) | 26.00 (24.21, 27.78) | 0.996 |
| TBA, M (Q₁, Q₃) | 5 | 1.55 (0.90, 2.54) | 1.55 (0.90, 2.55) | 0.999 |
| HbA1c, M (Q₁, Q₃) | 19 | 5.80 (5.60, 6.10) | 5.80 (5.60, 6.20) | 0.999 |
| Three-vessel artery disease, n (%), n(%) | 4 | 255 (30.36) | 254 (30.38) | 0.991 |

Abbreviations: BMI, Body Mass Index; HbA1c, Glycated hemoglobin; TBA, Total bile acids.

**Table S2**. Baseline characteristics of the training set and testing set.

| **Variables** | **Training set**  **(n = 672)** | **Testing set**  **(n = 168)** | ***P*** |
| --- | --- | --- | --- |
| Sex, n (%) |  |  | 0.609 |
| Female | 63 (9.4) | 13 (7.7) |  |
| Male | 609 (90.6) | 155 (92.3) |  |
| Age(years), (IQR) | 42 (38, 44) | 41 (37, 44) | 0.073 |
| BMI≥24 kg/m², n (%) | 513 (76.3) | 134 (79.8) | 0.401 |
| Smoke, n (%) | 480 (71.4) | 117 (69.6) | 0.718 |
| Alcohol, n (%) | 253 (37.7) | 69 (41.1) | 0.467 |
| Family history of CAD, n (%) | 78 (11.6) | 26 (15.5) | 0.218 |
| **Previous history** |  |  |  |
| Hypertension, n (%) | 337 (50.2) | 76 (45.2) | 0.293 |
| Diabetes, n (%) | 162 (24.1) | 34 (20.2) | 0.338 |
| Hyperlipidemia, n (%) | 180 (26.8) | 41 (24.4) | 0.597 |
| **Type of AMI, n (%)** |  |  |  |
| NSTEMI, n (%) | 184 (27.4) | 36 (21.4) | 0.141 |
| STEMI, n (%) | 488 (72.6) | 132 (78.6) | 0.141 |
| **Admission vital signs** |  |  |  |
| HR＞75bpm, n (%) | 80 (11.9) | 18 (10.7) | 0.768 |
| SBP＞140mmhg, n (%) | 665 (99.0) | 167 (99.4) | 1.000 |
| DBP＞90mmhg, n (%) | 153 (22.8) | 29 (17.3) | 0.149 |
| **Laboratory examination** |  |  |  |
| WBC＞10*10^9^/L, n (%) | 309 (46.0) | 80 (47.6) | 0.769 |
| NEU%＞75 %, n (%) | 262 (39.0) | 64 (38.1) | 0.901 |
| HCT＜45 %, n (%) | 452 (67.3) | 103 (61.3) | 0.172 |
| Hb＜120g/L, n (%) | 28 (4.2) | 5 (3.0) | 0.625 |
| PLT＞300*10^12^/L, n (%) | 102 (15.2) | 34 (20.2) | 0.140 |
| TBA＞1.56 umol/L, n (%) | 317 (47.2) | 78 (46.4) | 0.931 |
| TBil＞17.1 umol/L, n (%) | 183 (27.2) | 46 (27.4) | 1.000 |
| Cr＞110 umol/L, n (%) | 20 (3.0) | 2 (1.2) | 0.281 |
| UA＞360 umol/L, n (%) | 307 (45.7) | 85 (50.6) | 0.292 |
| HCY＞15umol/L, n (%) | 224 (33.3) | 56 (33.3) | 1.000 |
| Glu＞7.0 mmol/L, n (%) | 170 (25.3) | 42 (25.0) | 1.000 |
| HbA1C＞6.5 %, n (%) | 148 (22.0) | 28 (16.7) | 0.156 |
| TC＞5.20 mmol/L, n (%) | 239 (35.6) | 44 (26.2) | 0.027 |
| TG＞1.70 mmol/L, n (%) | 415 (61.8) | 110 (65.5) | 0.423 |
| LDL-C＞3.40 mmol/L, n (%) | 284 (42.3) | 55 (32.7) | 0.031 |
| HDL-C＜1.0mmol/L, n (%) | 473 (70.4) | 120 (71.4) | 0.865 |
| FFA＞0.9mmol/L, n (%) | 48 (7.14) | 17 (10.1) | 0.259 |
| CRP＞5.0 mg/L, n (%) | 340 (50.6) | 79 (47.0) | 0.458 |
| CLR＞2.83, n (%) | 347 (51.6) | 73 (43.5) | 0.070 |
| MLR＞0.3 n (%) | 340 (50.6) | 80 (47.6) | 0.546 |
| D-dimer＞0.5 mg/L, n (%) | 82 (12.2) | 28 (16.7) | 0.160 |
| Fg＞4.0 g/L, n (%) | 134 (19.9) | 27 (16.1) | 0.303 |
| CK-MB＞77 U/L, n (%) | 336 (50.0) | 77 (45.8) | 0.379 |
| cTnT＞1.44 ng/L, n (%) | 342 (50.9) | 78 (46.4) | 0.343 |
| BNP＞100 pg/ml, n (%) | 480 (71.4) | 121 (72.0) | 0.954 |
| **Coronary angiography** |  |  |  |
| SYNTAX Score＞14.5, n (%) | 330 (49.1) | 88 (52.4) | 0.501 |
| Three-vessel artery disease, n (%) | 211 (31.4) | 44 (26.2) | 0.223 |
| Left main artery disease, n (%) | 15 (2.2) | 4 (2.4) | 1.000 |
| Complete coronary occlusion, n (%) | 360 (53.6) | 92 (54.8) | 0.849 |

Abbreviations: BMI, Body Mass Index; BNP, Brain natriuretic peptide; CAD, Coronary artery disease; CK-MB, Creatine kinase isoenzyme; CLR, C-reactive Protein to Lymphocyte Ratio; cTnT, cardiac troponin T; Cr, Creatinine; CRP, C-reactive protein; DBP, Diastolic blood pressure; FFA, Free fatty acids; Fg, Fibrinogen; Glu, Glucose; Hb, Hemoglobin; HbA1c, Glycated hemoglobin; HCT, Hematocrit; HCY, Homocysteine; HDL-C, High-density lipoprotein cholesterol; HR, Heart rate; LDL-C, Low-density lipoprotein cholesterol; LVEF, Left ventricular ejection fraction; MLR, Monocyte to Lymphocyte Ratio; NEU%, Neutrophils percentage; NSTEMI, Non-ST-segment elevation myocardial infarction; PLT, Platelet; SBP, Systolic blood pressure; STEMI, ST-segment elevation myocardial infarction; TBA, Total bile acids; TBil, Total bilirubin; TC, Total cholesterol; TG, Triglyceride; UA, Uric acid; WBC, White blood cell.

**Table S3.** Baseline continuous variables of the HFpEF group and Non-HF group.

| **Variables** | **Total (n = 840)** | **Non-HF(N=572)** | **HFpEF(N=268)** | ***P*** |
| --- | --- | --- | --- | --- |
| Age, years, (IQR) | 42 (38, 44) | 41 (37, 44) | 42 (39, 45) | 0.001 |
| BMI, kg/m², (IQR) | 26 (24.22, 27.78) | 26 (23.9, 27.78) | 26 (24.5, 27.7) | 0.002 |
| HR, bpm, (IQR) | 73 (65, 82) | 72 (65, 81) | 75 (68, 85) | 0.578 |
| SBP, mmhg, (IQR) | 130 (120, 143) | 130 (120, 141) | 130 (118, 145) | 0.639 |
| DBP, mmhg, (IQR) | 80 (70, 90) | 80 (72, 90) | 80 (70, 90) | 0.020 |
| WBC, 109/L, (IQR) | 9.75 (8.08, 11.98) | 9.64 (7.81, 11.7) | 10.02 (8.6, 12.61) | 0.001 |
| NEU%, (IQR) | 72.3 (65.4, 78.9) | 71.1 (64.57, 78.2) | 74.65 (68.3, 80.3) | < 0.001 |
| HCT, %, (IQR) | 43.4 (40.8, 45.9) | 44 (41.18, 46.3) | 42.55 (39.9, 44.82) | < 0.001 |
| Hb, g/L, (IQR) | 147 (139, 157) | 149 (140, 158) | 144 (135, 154) | 0.561 |
| PLT, 1012/L, (IQR) | 238.5 (207, 279) | 239 (208, 275) | 238 (204.75, 285) | 0.246 |
| TBA, umol/L, (IQR) | 1.55 (0.9, 2.54) | 1.55 (0.96, 2.55) | 1.48 (0.76, 2.5) | 0.004 |
| Tbil, umol/L, (IQR) | 13.2 (9.6, 17.72) | 12.7 (9.3, 17.22) | 14.05 (10, 18.88) | 0.615 |
| Cr, umol/L, (IQR) | 74.5 (65, 84) | 74 (66, 83) | 76 (64, 86) | 0.003 |
| UA, umol/L, (IQR) | 355 (291, 420.25) | 359 (302, 426) | 341 (276.5, 410.75) | 0.246 |
| HCY, umol/L, (IQR) | 12.83 (10, 18.22) | 12.88 (10.19, 18.98) | 12.3 (9.9, 17.7) | 0.073 |
| Glu, mmol/L, (IQR) | 5.58 (4.95, 7.04) | 5.53 (4.92, 6.92) | 5.73 (5.04, 7.44) | 0.383 |
| HbA1c, %, (IQR) | 5.8 (5.6, 6.1) | 5.8 (5.6, 6.1) | 5.8 (5.5, 6.1) | 0.866 |
| TC, mmol/L, (IQR) | 4.78 (4.11, 5.5) | 4.8 (4.11, 5.5) | 4.78 (4.12, 5.5) | < 0.001 |
| TG, mmol/L, (IQR) | 2.03 (1.43, 2.98) | 2.12 (1.54, 3.15) | 1.81 (1.32, 2.56) | 0.197 |
| LDL-C, mmol/L, (IQR) | 3.16 (2.51, 3.78) | 3.13 (2.47, 3.75) | 3.23 (2.61, 3.82) | 0.005 |
| HDL-C, mmol/L, (IQR) | 0.91 (0.78, 1.05) | 0.9 (0.78, 1.02) | 0.93 (0.8, 1.1) | < 0.001 |
| FFA, mmol/L, (IQR) | 0.52 (0.5, 0.58) | 0.52 (0.48, 0.58) | 0.58 (0.58, 0.58) | < 0.001 |
| CRP, mg/L, (IQR) | 5 (2.13, 10.72) | 4.26 (1.8, 9.01) | 7.76 (3.16, 16.39) | < 0.001 |
| CLR, (IQR) | 2.83 (1.07, 6.44) | 2.2 (0.9, 4.83) | 4.43 (1.77, 12.47) | < 0.001 |
| MLR, (IQR) | 0.3 (0.23, 0.41) | 0.28 (0.22, 0.37) | 0.36 (0.27, 0.51) | < 0.001 |
| D-dimer, mg/L, (IQR) | 0.26 (0.21, 0.36) | 0.26 (0.2, 0.34) | 0.29 (0.23, 0.42) | < 0.001 |
| Fg, g/L, (IQR) | 3.24 (2.83, 3.8) | 3.21 (2.78, 3.69) | 3.41 (2.92, 4.06) | < 0.001 |
| CK-MB, U/L, (IQR) | 77 (28, 151.25) | 62 (24.75, 133.25) | 95.5 (39.75, 189.25) | < 0.001 |
| cTnT, ng/L, (IQR) | 1.44 (0.43, 3.57) | 1.15 (0.34, 3.03) | 2.33 (0.98, 4.61) | < 0.001 |
| BNP, pg/ml, (IQR) | 176 (85.43, 417.38) | 127 (63.56, 225.98) | 654.7 (349.59, 1026) | 0.001 |
| SYNTAX Score, (IQR) | 14.5 (9, 21) | 14 (9, 20) | 17 (11.75, 21.5) | 0.001 |

Abbreviations: BMI, Body Mass Index; BNP, Brain natriuretic peptide; CK-MB, Creatine kinase isoenzyme; CLR, C-reactive Protein to Lymphocyte Ratio; cTnT, cardiac troponin T; Cr, Creatinine; CRP, C-reactive protein; DBP, Diastolic blood pressure; FFA, Free fatty acids; Fg, Fibrinogen; Glu, Glucose; Hb, Hemoglobin; HbA1c, Glycated hemoglobin; HCT, Hematocrit; HCY, Homocysteine; HDL-C, High-density lipoprotein cholesterol; HR, Heart rate; LDL-C, Low-density lipoprotein cholesterol; MLR, Monocyte to Lymphocyte Ratio; NEU%, Neutrophils percentage; PLT, Platelet; SBP, Systolic blood pressure; TBA, Total bile acids; TBil, Total bilirubin; TC, Total cholesterol; TG, Triglyceride; UA, Uric acid; WBC, White blood cell.

**Figure S1** The distribution of data before and after SMOTE


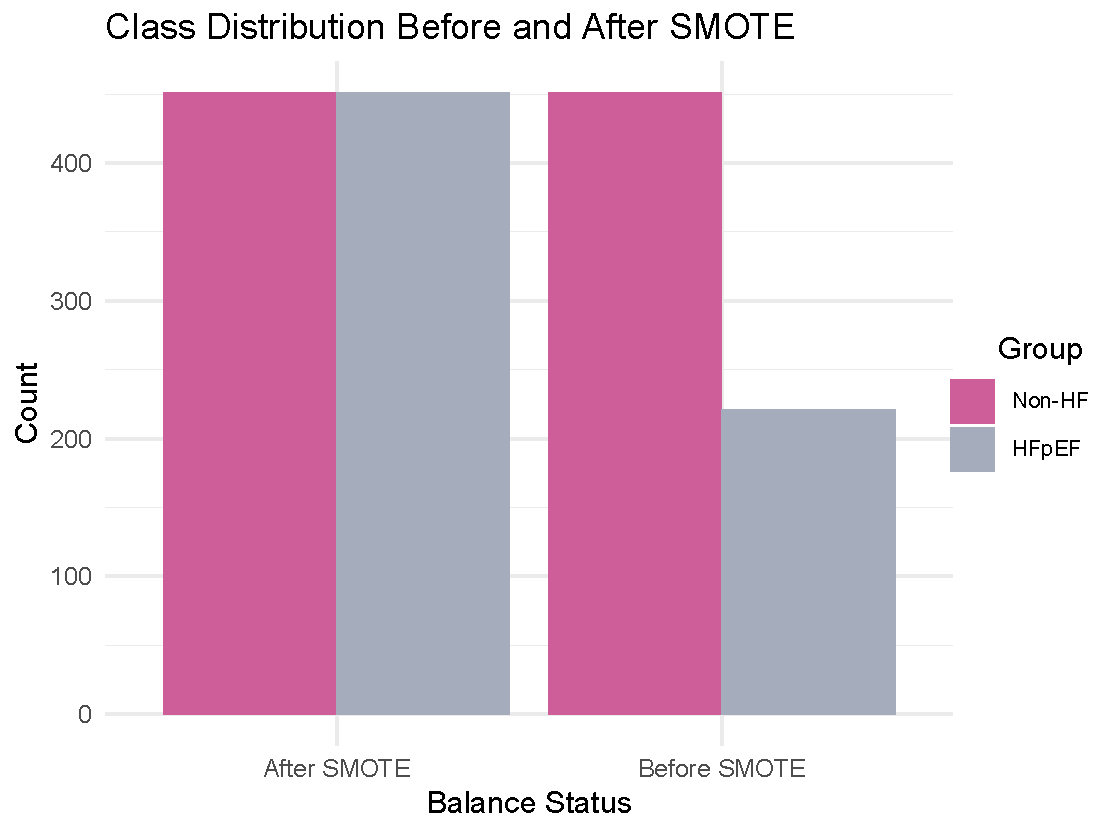


**Table S4.** Comparison of the Performance of Five Machine Learning Models Before and After Applying Synthetic Minority Over-sampling Technique (SMOTE).

| **Model** | **No-SMOTE** | | | | |  | **SMOTE** | | | | |
| --- | --- | --- | --- | --- | --- | --- | --- | --- | --- | --- | --- |
|  | **Precision** | **Accuracy** | **AUC** | **F1 score** | **Brier score** |  | **Precision** | **Accuracy** | **AUC** | **F1 score** | **Brier score** |
| **Lasso-Logistic** | 0.762 | 0.786 | 0.851 | 0.470 | 0.143 |  | 0.513 | 0.732 | 0.839 | 0.640 | 0.172 |
| **XGBoost** | 0.686 | 0.798 | 0.854 | 0.586 | 0.143 |  | 0.643 | 0.815 | 0.897 | 0.699 | 0.123 |
| **RF** | 0.667 | 0.756 | 0.846 | 0.369 | 0.155 |  | 0.556 | 0.768 | 0.842 | 0.673 | 0.169 |
| **KNN** | 0.516 | 0.726 | 0.726 | 0.410 | 0.177 |  | 0.488 | 0.714 | 0.737 | 0.455 | 0.186 |
| **SVM** | 0.280 | 0.280 | 0.517 | 0.438 | 0.204 |  | 0.275 | 0.234 | 0.485 | 0.429 | 0.251 |

**Table S5.** Net Reclassification Improvement (NRI）and Integrated Discrimination Improvement (IDI）of the XGBoost model compared with other models

| **Models** | **NRI [95% CI]** | ***P*** | **IDI [95% CI]** | ***P*** |
| --- | --- | --- | --- | --- |
| **Lasso-Logistic** | 0.149[0.008-0.290] | 0.039 | 0.049[0.008- 0.091] | 0.019 |
| **RF** | 0.222[0.067-0.377] | 0.005 | 0.051[0.015-0.086] | 0.005 |
| **KNN** | 0.212[0.028-0.396] | 0.025 | 0.067[0.004-0.130] | 0.038 |
| **SVM** | 0.428[0.277-0.579] | ＜0.001 | 0.227[0.175-0.279] | ＜0.001 |

**Table S6.** Logistic regression analysis between the variables and HFpEF

| **Variables** | **OR (95%CI)** | ***P*** |
| --- | --- | --- |
| Male, n (%) | 0.34 (0.21 ~ 0.55) | <0.001 |
| Age (years) Median (Q1, Q3) | 1.05 (1.02 ~ 1.08) | 0.001 |
| BMI≥24 kg/m², n (%) | 1.61 (1.12 ~ 2.33) | 0.011 |
| Smoke, n (%) | 0.76 (0.56 ~ 1.04) | 0.088 |
| Alcohol, n (%) | 1.01 (0.75 ~ 1.36) | 0.968 |
| Family history of CAD, n (%) | 0.80 (0.51 ~ 1.27) | 0.348 |
| **Previous history** |  |  |
| Hypertension, n (%) | 1.53 (1.14 ~ 2.05) | 0.005 |
| Diabetes, n (%) | 1.05 (0.74 ~ 1.47) | 0.797 |
| Hyperlipidemia, n (%) | 0.88 (0.63 ~ 1.23) | 0.449 |
| **Type of AMI, n (%)** |  |  |
| STEMI | 1.73 (1.21 ~ 2.46) | 0.002 |
| **Admission vital signs** |  |  |
| HR＞75bpm, n (%) | 2.17 (1.42 ~ 3.33) | <0.001 |
| SBP＞140mmhg, n (%) | 0.78 (0.18 ~ 3.28) | 0.734 |
| DBP＞90mmhg, n (%) | 1.03 (0.73 ~ 1.46) | 0.867 |
| **Laboratory examination** |  |  |
| WBC＞10*10^9^/L, n (%) | 1.27 (0.95 ~ 1.70) | 0.106 |
| NEU%＞75 %, n (%) | 1.81 (1.35 ~ 2.43) | <0.001 |
| HCT＜45 %, n (%) | 1.95 (1.41 ~ 2.70) | 0.002 |
| Hb＜120 g/L, n (%) | 1.95 (1.41 ~ 2.70) | <.001 |
| PLT＞300*10^12^/L, n (%) | 1.15 (0.78 ~ 1.70) | 0.468 |
| TBA＞1.56 umol/L, n (%) | 0.96 (0.72 ~ 1.28) | 0.764 |
| TBil＞17.1 umol/L, n (%) | 1.34 (0.98 ~ 1.85) | 0.069 |
| Cr＞110 umol/L, n (%) | 2.18 (0.93 ~ 5.10) | 0.071 |
| UA＞360 umol/L, n (%) | 0.77 (0.57 ~ 1.03) | 0.074 |
| HCY＞15umol/L, n (%) | 0.99 (0.73 ~ 1.35) | 0.958 |
| Glu＞7.0 mmol/L, n (%) | 1.13 (0.81 ~ 1.58) | 0.457 |
| HbA1c＞6.5 %, n (%) | 1.06 (0.75 ~ 1.51) | 0.737 |
| TC＞5.20 mmol/L, n (%) | 0.99 (0.73 ~ 1.35) | 0.958 |
| TG＞1.70 mmol/L, n (%) | 0.99 (0.73 ~ 1.35) | 0.964 |
| LDL-C＞3.40 mmol/L, n (%) | 0.90 (0.67 ~ 1.21) | 0.491 |
| HDL-C＜1.0 mmol/L, n (%) | 1.14 (0.85 ~ 1.53) | 0.378 |
| FFA＞0.9 mmol/L, n (%) | 0.77 (0.56 ~ 1.05) | 0.098 |
| CRP＞5.0 mg/L, n (%) | 1.47 (0.87 ~ 2.47) | 0.147 |
| CLR＞2.83, n (%) | 2.45 (1.82 ~ 3.31) | <0.001 |
| MLR＞0.3 n (%) | 2.57 (1.90 ~ 3.48) | <0.001 |
| D-dimer＞0.5 mg/L, n (%) | 1.79 (1.19 ~ 2.70) | 0.005 |
| Fg＞4.0 g/L, n (%) | 1.81 (1.27 ~ 2.58) | 0.001 |
| CK-MB＞77 U/L, n (%) | 1.63 (1.22 ~ 2.19) | 0.001 |
| cTnT＞1.44 ng/L, n (%) | 1.99 (1.48 ~ 2.68) | <0.001 |
| BNP＞100 pg/ml, n (%) | 6.12 (3.90 ~ 9.62) | <0.001 |
| **Coronary angiography** |  |  |
| SYNTAX Score＞14.5, n (%) | 2.37 (1.76 ~ 3.20) | <0.001 |
| Three-vessel artery disease, n (%) | 1.13 (0.82 ~ 1.54) | 0.455 |
| Left main artery disease, n (%) | 2.42 (0.97 ~ 6.04) | 0.057 |
| Complete coronary occlusion, n (%) | 1.16 (0.87 ~ 1.56) | 0.313 |

Abbreviations: ACEI, Angiotensin-converting enzyme inhibitor; ARB, Angiotensin-receptor blocker; ARNI, Angiotensin receptor-neprilysin inhibitor; BMI, Body Mass Index; BNP, Brain natriuretic peptide; CAD, Coronary artery disease; CK-MB, Creatine kinase isoenzyme; CLR, C-reactive Protein to Lymphocyte Ratio; cTnT, cardiac troponin T; Cr, Creatinine; CRP, C-reactive protein; DBP, Diastolic blood pressure; FFA, Free fatty acids; Fg, Fibrinogen; Glu, Glucose; Hb, Hemoglobin; HbA1c, Glycated hemoglobin; HCT, Hematocrit; HCY, Homocysteine; HDL-C, High-density lipoprotein cholesterol; HR, Heart rate; LDL-C, Low-density lipoprotein cholesterol; LVEF, Left ventricular ejection fraction; MLR, Monocyte to Lymphocyte Ratio; MRA, Mineralocorticoid receptor antagonist; NEU%, Neutrophils percentage; NSTEMI, Non-ST-segment elevation myocardial infarction; PLT, Platelet; SBP, Systolic blood pressure; STEMI, ST-segment elevation myocardial infarction; TBA, Total bile acids; TBil, Total bilirubin; TC, Total cholesterol; TG, Triglyceride; UA, Uric acid; WBC, White blood cell.
